# Early risk stratification in lupus nephritis based on established prognostic factors: association with outcomes under triple therapy

**DOI:** 10.1186/s13075-026-03876-w

**Published:** 2026-07-31

**Authors:** Hironari Hanaoka, Ryota Sakai, Takumi Aoki, Yuri Sasaki, Taiji Kosaka, Shoichi Yoshinaga, Akiko Shibata, Takahiko Kurasawa, Koichi Amano

**Affiliations:** Department of Rheumatology and Clinical Immunology, Saitama Medical Center, Saitama Medical University, 1981 Kamoda, Kawagoe, Saitama, 350-8550 Japan

**Keywords:** Lupus nephritis, Triple therapy, Prognostic factor, Complete renal response, Risk stratification

## Abstract

**Background:**

To apply the 2025 EULAR prognostic factors to identify subgroups of patients with lupus nephritis (LN) in which triple therapy was associated with higher complete renal response (CR) rates.

**Methods:**

We conducted a medical record review of patients with biopsy-proven class III, IV, or V LN who were treated at our hospital between 2000 and 2024. Multivariate logistic regression was used to identify EULAR 2025 prognostic factors independently associated with CR. Patients were stratified according to major prognostic factors and the cumulative burden of additional factors. Cumulative CR rates were compared, and Cox proportional hazards models were applied to evaluate the association between risk stratification and CR.

**Results:**

Among the 170 patients included in this study, nephrotic-range proteinuria and a history of previous LN flare were negatively associated with CR (odds ratio [OR] 0.25, 95% confidence interval [CI] 0.11–0.55, *p* < 0.01 and OR 0.29, 95% CI 0.12–0.65, *p* < 0.01, respectively). Using a stepwise risk stratification approach incorporating these factors and the cumulative number of remaining prognostic factors, patients were classified into low-, moderate-, and high-risk groups, showing a clear gradient in CR rates (94.0% vs. 71.8% vs. 40.9%, respectively, *p* < 0.01). Using the high-risk group as the reference, the likelihood of achieving CR was substantially higher in the low- and moderate-risk groups (hazard ratio [HR] 22.8, 95% CI 8.27–81.24, p < 0.01 and HR 3.7, 95% CI 1.53–9.52, *p* = 0.01). In the high-risk group, triple therapy remained independently associated with CR (adjusted HR 3.13, 95% CI 1.17–8.73, *p* = 0.02).

**Conclusions:**

These findings suggest that baseline risk stratification identified subgroups in which triple therapy was associated with higher CR rates. Prospective validation in independent cohorts is required before this approach can inform treatment selection.

**Supplementary Information:**

The online version contains supplementary material available at https://doi.org/10.1186/s13075-026-03876-w.

## Background

Lupus nephritis (LN) remains one of the most severe organ manifestations of systemic lupus erythematosus (SLE) and a major determinant of long-term morbidity and mortality [1–5]. Over the past decade, therapeutic strategies for LN have evolved substantially, along with an improved understanding of early treatment response as a predictor of long-term renal outcomes [6, 7].

The 2024 American College of Rheumatology (ACR) guideline recommends triple therapy, glucocorticoids, mycophenolate mofetil (MMF), and either a calcineurin inhibitor (CNIs) or belimumab, as the standard induction treatment for patients with class III/IV ± V LN [8]. Recently, the 2025 update of the EULAR recommendations for the management of SLE with kidney involvement has further emphasized the early use of combination therapy, particularly in patients with poor prognostic features [9]. Importantly, these recommendations acknowledge that single-agent or dual therapies remain an option for patients without high-risk characteristics. Although LN is a potentially devastating complication, it is evident that a subset of patients with favorable baseline characteristics can achieve sustained remission with less-intensive regimens [10]. Therefore, the universal application of triple therapy may expose low-risk patients to unnecessary immunosuppressive burdens, increased toxicity, and substantial economic costs. Consequently, baseline risk stratification using readily available clinical and histopathological factors is critical to determine the appropriate intensity of induction therapy.

Although the 2025 EULAR recommendations summarize several established adverse prognostic factors for LN, these factors have not been systematically integrated into a clinically applicable risk stratification framework [9]. The present study applied the EULAR 2025 prognostic factors to stratify patients with LN according to baseline risk and identify patient subsets in which triple therapy was associated with higher CR rates.

## Methods

### Patients

We conducted a medical record review of patients with SLE who fulfilled the EULAR/ACR classification criteria [11] and were treated at Saitama Medical Center, Saitama Medical University Hospital, between January 2000 and December 2024. Among these patients, those with biopsy-proven class III, IV, or V LN were classified according to the International Society of Nephrology/Renal Pathology Society (ISN/RPS) criteria [12] and with a minimum follow-up period of three years were eligible for inclusion. Only the first induction episodes during the study period were included. This study was approved by the Ethics Committee of Saitama Medical Center, Saitama Medical University (approval number: 2025–146) and conformed to the ethical principles of the Declaration of Helsinki (2024). Owing to the observational design based on medical record review and exclusive use of clinical data obtained during routine medical care, the requirement for written informed consent was waived in accordance with the Ethical Guidelines for Medical and Health Research Involving Human Subjects issued by the Japanese Ministry of Health, Labour, and Welfare.

### Data collection and clinical outcomes

Clinical data were collected at baseline; three and six months; and one, two, and three years following the initiation of induction therapy. The baseline variables were defined as those obtained at the time of renal biopsy before treatment initiation. Data were collected on demographic characteristics, laboratory parameters, treatment regimens, and disease activity were assessed using the Systemic Lupus Erythematosus Disease Activity Index (SLEDAI) [13]. The primary outcome was complete renal response (CR). CR was defined according to the EULAR recommendations for LN [9, 14] as a urine protein-to-creatinine ratio (UPCR) ≤ 0.5 g/gCr accompanied by stabilization of estimated glomerular filtration rate (eGFR) to > 80% of baseline value. In addition, we used the Systemic Lupus International Collaborating Clinics/American College of Rheumatology Damage Index (SDI) to assess the systemic damage accrual [15].

Triple therapy was defined as the concomitant use of glucocorticoids, tacrolimus, and either MMF or pulsed cyclophosphamide initiated within 1 month of the induction treatment.

### Renal pathology

All patients underwent renal biopsy before the initiation of induction therapy. Paraffin-embedded specimens were examined using light microscopy with hematoxylin–eosin (HE), Masson’s trichrome, periodic acid–Schiff, and periodic acid methenamine silver staining. Immunofluorescence analysis was performed on the frozen sections using fluorescent antisera against IgG, IgA, IgM, C3, C4, C1q, and fibrinogen. The LN class was assigned according to the ISN/RPS classification [12]. The activity index (AI) and chronicity index (CI) were calculated as previously scored [4]. Individual histopathological components, including endocapillary hypercellularity, leukocyte infiltration, karyorrhexis/fibrinoid necrosis, cellular crescents, hyaline deposits, interstitial inflammation, glomerular sclerosis, fibrous crescents, tubular atrophy, and interstitial fibrosis, were assessed, and the proportion of involved glomeruli or interstitial areas was recorded.

### Risk assessment at initial therapy

Baseline risk stratification was developed using established prognostic factors summarized in the 2025 EULAR recommendations for LN, including nephrotic-range proteinuria, previous LN flare, baseline eGFR < 60 mL/min/1.73 m^2^, male sex, hypertension, chronicity index > 3, histological crescents, and severe tubulointerstitial inflammation [9]. The prognostic factors and corresponding thresholds were based on the 2025 EULAR recommendations. However, the classification of patients into low-, moderate-, and high-risk categories was pre-specified a priori for the purposes of this study and does not represent a validated EULAR risk stratification system. Severe tubulointerstitial inflammation was defined as the involvement of > 50% of the tubulointerstitial area on renal biopsy. Multivariate logistic regression analysis was conducted to identify prognostic factors independently associated with CR achievement. Prognostic factors independently associated with CR in the multivariable logistic regression analysis were designated as major risk factors. The remaining established EULAR prognostic factors, which did not retain independent statistical significance after multivariable adjustment, were classified as minor risk factors. The patients were initially stratified according to the presence or absence of major risk factors and subsequently subdivided based on the cumulative number of minor risk factors. CR rates were compared across these subgroups. To further evaluate the prognostic performance of this risk stratification, Cox proportional hazards regression analysis was performed to estimate the hazard ratios (HRs) for achieving CR.

### Statistical analysis

Continuous variables are presented as mean ± standard deviation (SD). Differences between groups were analyzed using the Mann–Whitney *U*-test or Kruskal–Wallis test for nonparametric data and the chi-squared or Fisher’s exact test for categorical data. Cumulative CR rates were estimated using the Kaplan–Meier method and compared using the log-rank test. Multivariate logistic regression analysis was conducted to identify the EULAR prognostic factors independently associated with CR. To evaluate whether triple therapy was independently associated with CR achievement among high-risk patients classified as high risk, multivariable Cox proportional hazards regression analysis was performed. Triple therapy was the primary explanatory variable. Covariates were selected a priori based on their established clinical relevance, potential influence on both treatment allocation and renal outcomes, and the need to avoid overadjustment and multicollinearity in the relatively small high-risk subgroup. Accordingly, baseline UPCR, disease duration, and eGFR were included in the multivariate Cox model. The adjusted HRs with 95% confidence intervals (CIs) were calculated. All statistical analyses were performed using GraphPad Prism, version 7 (GraphPad Software, San Diego, CA, USA). Statistical significance was set at *p* < 0.05 (two-sided).

## Results

### Baseline clinicopathological characteristics and treatment regimens

In total, 170 patients with biopsy-proven class III, IV, or V LN were included in the analysis (Table 1). The cohort was predominantly female (81.7%), with a mean age of 39.4 years and a mean disease duration of 74.3 months. At baseline, the mean UPCR was 3.7 g/gCr and the mean eGFR was 69.3 mL/min/1.73 m^2^. Induction therapy was initiated with a mean prednisolone dose of 0.82 mg/kg/d. Regarding immunosuppressive regimens, 37.6% of the patients received triple therapy, whereas 62.4% received dual therapy. Hydroxychloroquine was used in 32.9% of patients; this relatively low proportion was largely attributable to the fact that HCQ was not approved for the treatment of SLE in Japan until 2015.

**Table 1:** Baseline clinical features

Clinical feature	All patients (*n* = 170)	Low Risk (*n* = 67)	Moderate risk (*n* = 32)	High risk (*n* = 71)	*p*
Sex (female), n (%)	139.0 (81.7)	61.0 (91.1)	28.0 (87.5)	50.0 (70.4)	0.004
Age (years)	39.4 ± 14.4	35.1 ± 13.1	38.8 ± 18.2	43.7 ± 12.6	0.002
Disease duration (months)	74.3 ± 94.1	27.9 ± 55.1	68.9 ± 92.7	120.6 ± 102.4	< 0.001
BMI (kg/m^2^)	22.6 ± 4.9	21.5 ± 4.5	22.5 ± 4.2	23.7 ± 5.5	0.035
Dyslipidemia, n (%)	23.0 (13.5)	1.0 (1.5)	2.0 (6.3)	20.0 (28.2)	< 0.001
Diabetes mellitus, n (%)	24.0 (14.1)	2.0 (2.9)	12.0 (37.5)	10.0 (14.1)	< 0.001
Smoking ever, n (%)	50.0 (29.4)	14.0 (20.9)	10.0 (31.2)	26.0 (36.6)	0.124
Proteinuria (g/gCr)	3.7 ± 3.4	1.6 ± 1.2	3.6 ± 2.7	5.6 ± 3.9	< 0.001
eGFR (mL/min/1.73m^2^)	69.3 ± 30.1	78.6 ± 31.5	76.6 ± 32.9	59.7 ± 25.3	0.002
Anti-dsDNA antibody (IU/mL)	630.0 ± 1174.9	1000.3 ± 1376.9	404.3 ± 936.5	379.9 ± 967.6	0.003
Anti-cardiolipin antibody, n (%)	81.0 (47.6)	36.0 (53.7)	13.0 (40.6)	32.0 (45.1)	0.403
Anti-Sm antibody positive, n (%)	80.0 (47.1)	38.0 (56.7)	15.0 (46.9)	27.0 (38.0)	0.089
CH50 (U/mL)	25.3 ± 18.0	20.7 ± 16.8	28.6 ± 20.3	28.5 ± 17.4	0.033
SLEDAI	21.2 ± 7.6	23.5 ± 6.4	19.9 ± 7.1	19.6 ± 8.4	0.821
Statin use, n (%)	67 (39.4)	18 (26.9)	13 (40.6)	36 (50.7)	0.016
ACEi or ARB use, n (%)	103 (60.6)	28 (41.8)	21 (65.6)	54 (76.1)	< 0.001
Prednisolone (mg/kg/d)	0.82 ± 0.32	0.88 ± 0.24	0.83 ± 0.36	0.77 ± 0.33	0.133
Glucocorticoid pulse therapy, n (%)	41 (24.1)	17 (25.3)	7 (21.8)	17 (23.9)	0.929
HCQ use, n (%)	55.0 (32.4)	28.0 (41.8)	6.0 (18.8)	21.0 (29.6)	0.068
Triple therapy, n (%)	64.0 (37.6)	23.0 (34.3)	11.0 (34.4)	30.0 (42.3)	0.576
MMF + TAC, n (%)	14.0 (8.2)	5.0 (7.5)	2.0 (6.3)	7.0 (9.9)	0.792
pCYC + TAC, n (%)	40.0 (23.5)	15.0 (22.4)	8.0 (25.0)	17.0 (23.9)	0.954
Others, n (%)	9.0 (5.8)	3.0 (4.4)	1.0 (3.1)	5.0 (7.0)	0.663
Dual therapy, n (%)	106.0 (54.1)	46.0 (68.7)	21.0 (65.6)	39.0 (54.9)	0.298
pCYC, n (%)	42.0 (24.7)	20.0 (29.9)	6.0 (18.8)	16.0 (22.5)	0.418
MMF, n (%)	27.0 (15.9)	11.0 (16.4)	5.0 (15.6)	11.0 (15.5)	0.998
TAC, n (%)	18.0 (10.6)	8 (11.9)	4.0 (12.5)	6.0 (8.4)	0.352
Others, n (%)	19.0 (13.5)	7.0 (10.4)	6.0 (18.8)	6.0 (8.4)	0.623
Renal pathological findings			-	-	-
ISN/RPS classification					
III (A) or III (A/C) (%)	35.0 (20.6)	18.0 (26.9)	9.0 (28.1)	8.0 (11.3)	0.059
III (A) or III (A/C) + V (%)	13.0 (7.6)	5.0 (7.5)	1.0 (3.1)	7.0 (9.9)	0.491
IV (A) or IV (A/C) (%)	73.0 (42.9)	32.0 (47.8)	13.0 (40.6)	28.0 (39.4)	0.588
IV (A) or IV (A/C) + V (%)	37.0 (21.8)	10.0 (14.9)	7.0 (21.8)	20.0 (28.1)	0.169
Pure V (%)	12.0 (7.1)	2.0 (2.9)	2.0 (6.3)	8.0 (11.3)	0.162
AI	5.9 ± 3.7	5.6 ± 3.5	5.6 ± 4.2	6.6 ± 3.7	0.281
CI	1.4 ± 1.7	0.7 ± 1.1	1.1 ± 1.1	2.2 ± 2.1	< 0.001
Prognostic factor					
None, n (%)	49.0 (28.8)	49.0 (73.1)	0.0 (0.0)	0.0 (0.0)	< 0.001
Previous flare, n (%)	51.0 (30.0)	0.0 (0.0)	7.0 (21.8)	44.0 (61.9)	< 0.001
Nephrotic-range proteinuria, n (%)	70.0 (41.2)	0.0 (0.0)	17.0 (53.1)	53.0 (74.6)	< 0.001
Hypertension, n (%)	39.0 (22.9)	4.0 (5.9)	5.0 (15.6)	30.0 (42.3)	< 0.001
eGFR < 60 mL/min/1.73m^2^, n (%)	51.0 (30.0)	7.0 (10.4)	4.0 (12.5)	40.0 (56.3)	< 0.001
Male, n (%)	31.0 (18.2)	6.0 (8.9)	4.0 (12.5)	21.0 (29.6)	0.004
Crescent, n (%)	28.0 (16.4)	1.0 (1.5)	10.0 (31.2)	17.0 (23.9)	< 0.001
CI > 3.0, n (%)	31.0 (18.2)	0.0 (0.0)	4.0 (12.5)	27.0 (38.0)	< 0.001
Tubulointerstitial inflammation > 50%, n (%)	12.0 (7.1)	0.0 (0.0)	5.0 (15.6)	7.0 (9.8)	0.008
SDI at baseline	0.3 ± 0.7	0.1 ± 0.2	0.1 ± 0.2	0.7 ± 0.9	< 0.001
SDI at 12 months	0.6 ± 0.9	0.2 ± 0.5	0.3 ± 0.5	1.0 ± 1.2	< 0.001
SDI at 24 months	0.7 ± 1.1	0.2 ± 0.6	0.4 ± 0.7	1.2 ± 1.5	< 0.001
SDI at 36 M	0.8 ± 1.3	0.3 ± 0.8	0.5 ± 0.8	1.3 ± 1.7	< 0.001

*BMI* Body mass index, *HCQ* Hydroxychloroquine, *eGFR* Glomerular filtration rate, *SLEDAI* Systemic Lupus Erythematosus Disease Activity Index, *ACEi* Angiotensin-converting enzyme inhibitor, *ARB* Angiotensin II receptor blocker, *dsDNA* double-stranded DNA, *MMF* Mycophenolate mofetil, *TAC* Tacrolimus, *pCYC* pulsed cyclophosphamide, *SDI* Systemic Lupus Erythematosus Damage Index. Class IV (A) indicated diffuse active LN, whereas class IV (A/C) indicated diffuse active and chronic LN. The AI ranged 0–24 and the CI ranged 0–12

The most frequent histological subtype was ISN/RPS class IV (A or A/C), accounting for 42.9% of cases. The mean AI and CI were 5.9 and 1.4, respectively. Overall, 71.2% of patients had at least one EULAR 2025 prognostic factor. The most prevalent factors were nephrotic-range proteinuria (41.2%), previous LN flares (30.0%), and reduced baseline eGFR (< 60 mL/min/1.73 m^2^) (30.0%).

### Identification of major prognostic factors for CR

Multivariate logistic regression analysis incorporating the eight EULAR 2025 prognostic factors identified nephrotic-range proteinuria (odds ratio [OR] 0.25, 95% CI 0.11–0.55, *p* = 0.0005) and a history of previous LN flare (OR 0.29, 95% CI 0.12–0.65, *p* = 0.0028) as independent negative predictors of CR (Table 2). Based on these results, nephrotic-range proteinuria and previous LN flares were defined as major risk factors, whereas the remaining EULAR prognostic factors were classified as minor risk factors.

**Table 2:** Prognostic factors associated with CR

	**Odds ratio**	**95% CI**	***p***
Previous LN flare	0.29	0.12–0.65	< 0.01
Nephrotic-range proteinuria	0.25	0.11–0.55	< 0.01
Hypertension	0.41	0.16–0.99	0.06
eGFR < 60 mL/min/1.73m^2^	0.50	0.23–1.23	0.14
CI > 3.0	0.31	0.10–1.01	0.05
Male	0.69	0.25–1.92	0.47
Crescent	0.70	0.10–1.33	0.23
Severe tubulointerstitial inflammation	0.33	0.10–1.03	0.14

*CR* Complete renal response, *eGFR* Glomerular filtration rate

### Risk stratification based on cumulative CR rates

To assess the cumulative impact of prognostic factors on treatment response, Kaplan–Meier analysis was performed according to the combinations of major and minor risk factors (Supplementary Fig. 1). Patients with no prognostic factors and those with a single minor risk factor showed comparable cumulative CR rates (91.8% vs. 94.5%,* p* = 0.31). In contrast, the presence of at least one major risk factor was associated with a significantly lower CR rate (79.2% vs. 91.9%, *p* = 0.03). Patients with one major risk factor and those with two minor risk factors showed similar CR trajectories (79.2% vs. 50.0%; *p* = 0.19). However, patients with at least one major risk factor in combination with two or more prognostic factors exhibited a markedly reduced cumulative CR rate compared to patients without any risk factors (43.7% vs. 91.9%, *p* < 0.001), indicating a strong cumulative effect of adverse baseline features.

### Three-tier risk classification and association with CR

**Fig. 1:**
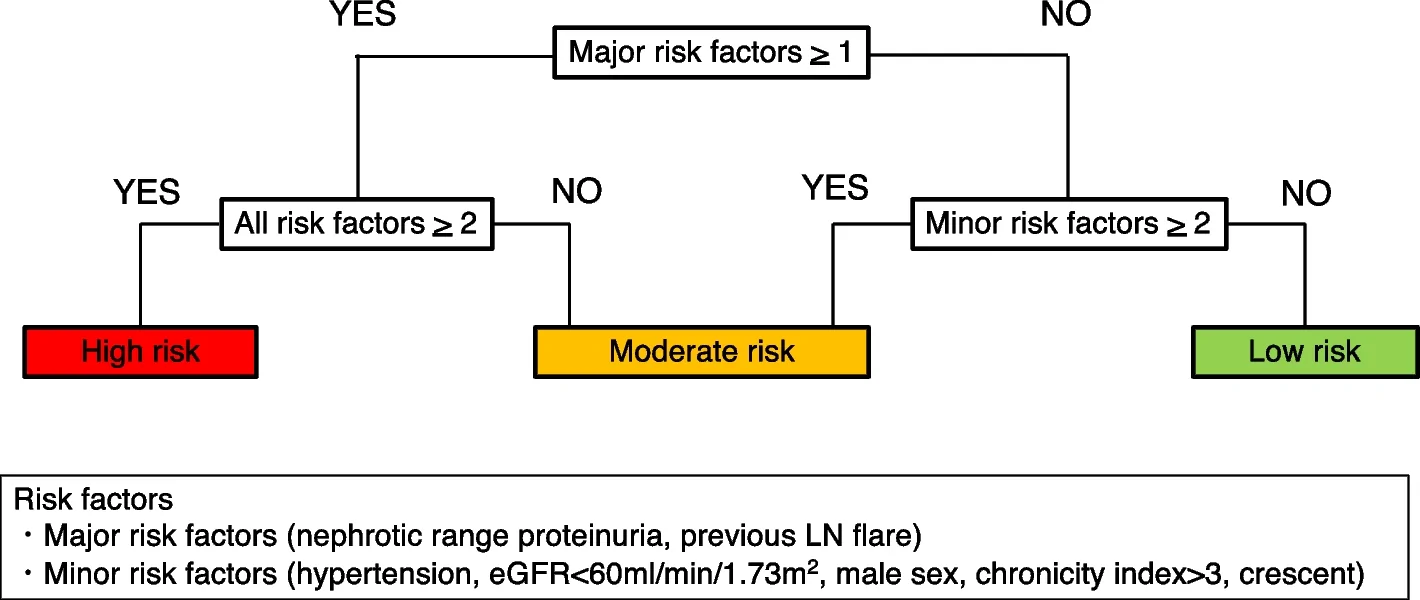
Risk-assessment algorithm for LN. Risk assessment algorithm for the baseline stratification of patients with LN into low-, moderate-, and high-risk categories based on prognostic factors. LN, lupus nephritis; eGFR, estimated glomerular filtration rate, CI, chronicity index; TI, tubulointerstitial inflammation

**Fig. 2:**
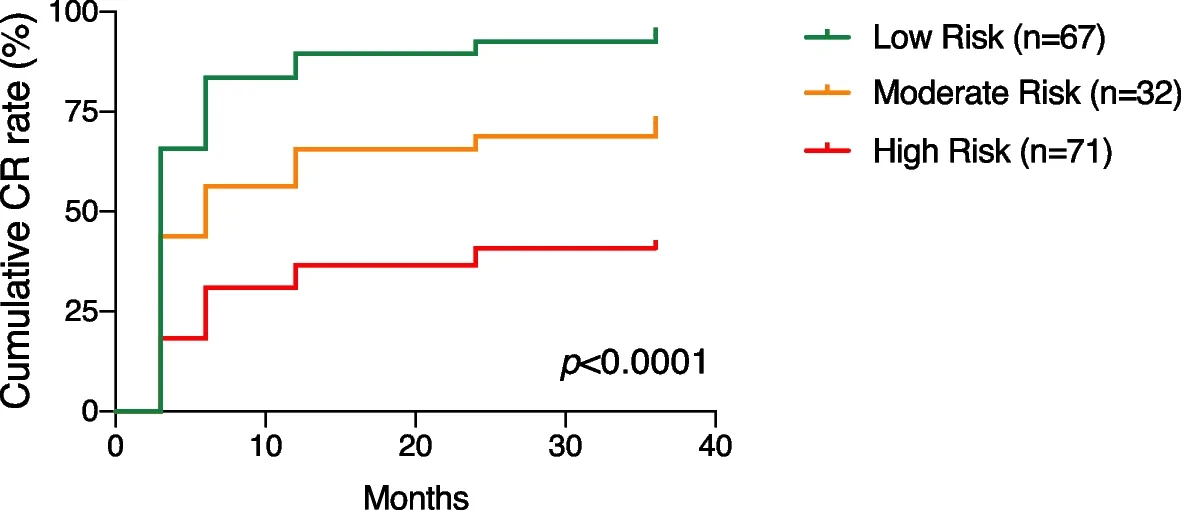
Cumulative CR rates according to baseline risk category. Kaplan–Meier curves showing cumulative CR rates according to the baseline risk category (low, moderate, and high risk), revealing significantly distinct remission trajectories across strata. CR: complete renal response

Based on these findings, a risk assessment algorithm was constructed to classify patients into three baseline risk categories: low- (*n* = 67, 39.4%), moderate- (*n* = 32, 18.8%), and high-risk (*n* = 71, 41.8%) (Fig. 1). The cumulative CR rates differed significantly across these strata, with 94.0% in the low-risk group, 71.8% in the moderate-risk group, and 40.9% in the high-risk group, respectively (*p* < 0.01) (Fig. 2).

**Fig. 3:**
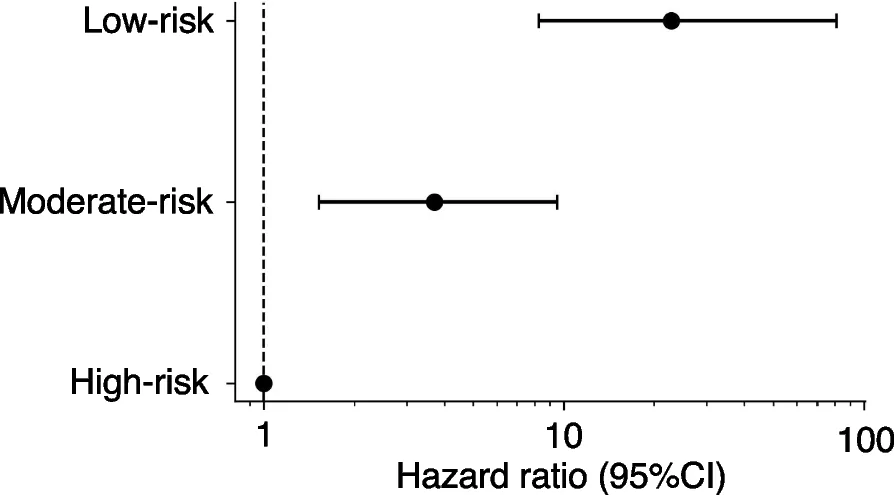
Prognostic impact of baseline risk classification on CR. Forest plot showing HRs for achievement of CR according to the baseline risk category based on Cox proportional hazards regression analysis, with the high-risk group as reference. CR: complete renal response

The association between risk classification and CR was further evaluated using the Cox proportional hazards regression analysis (Fig. 3). In the Cox proportional hazards analysis performed in the entire cohort, including both dual- and triple-therapy groups, and adjusted for baseline UPCR, disease duration, and eGFR, the probability of achieving CR was significantly higher in the low-risk group than in the high-risk group (hazard ratio [HR] 22.8, 95% CI 8.27–81.24, *p* < 0.01), and remained significantly higher in the moderate-risk group (HR 3.7, 95% CI 1.53–9.52, *p* = 0.01). In the high-risk group, triple therapy remained independently associated with CR (adjusted HR 3.13, 95% CI 1.17–8.73, *p* = 0.02).

### Clinical characteristics across risk groups

A comparison of the baseline clinicopathological features across the three risk categories revealed progressive enrichment of disease severity in the high-risk group (Table 1). High-risk patients had a higher proportion of males (29.6%), greater baseline UPCR (5.6 g/gCr), lower eGFR (59.7 mL/min/1.73 m^2^), and higher AI and CI (6.6 and 2.2, respectively), supporting the biological plausibility of the proposed stratification.

### Differential efficacy of triple therapy according to risk category

**Fig. 4:**
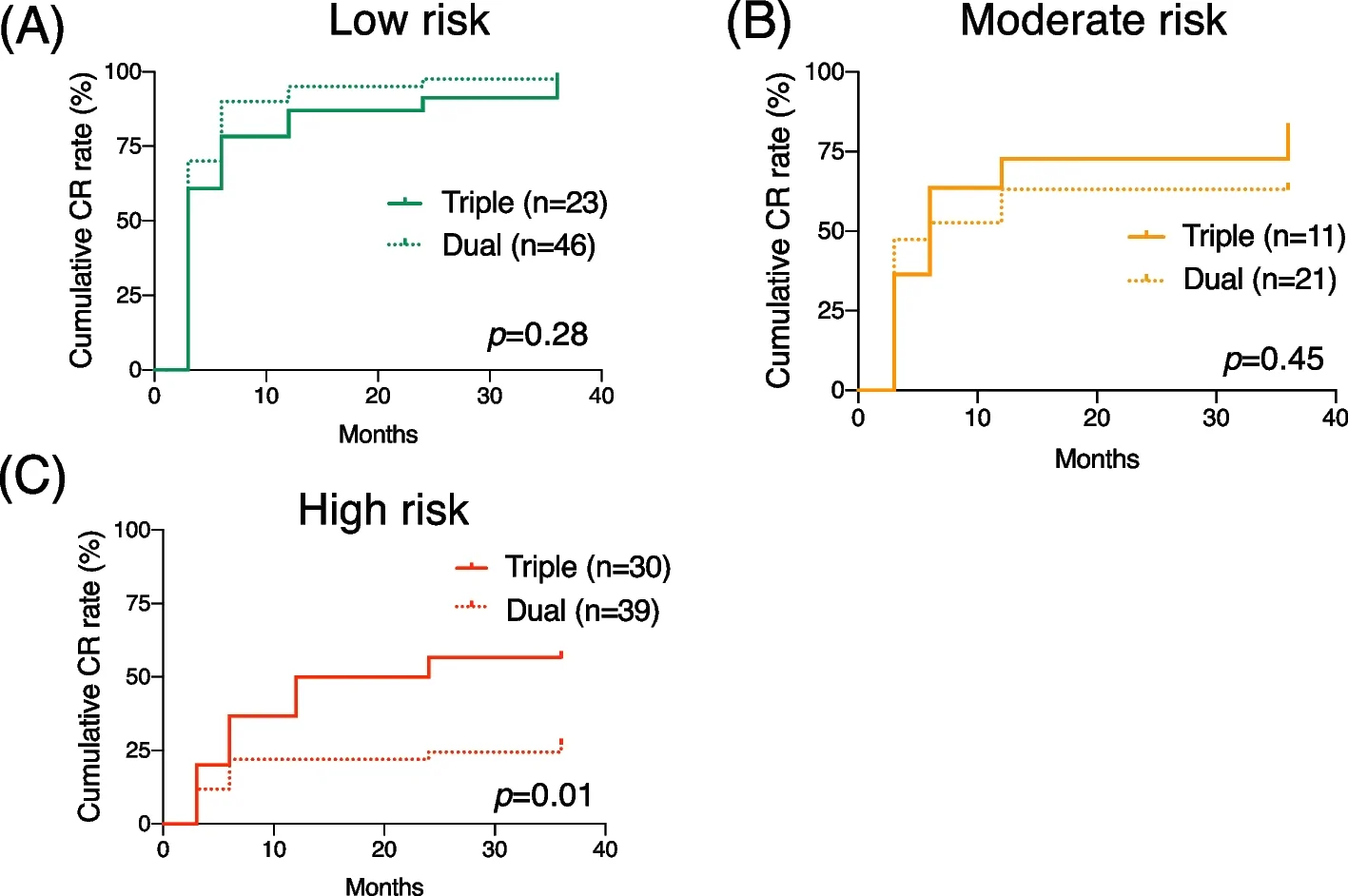
Differential efficacy of triple versus dual therapy according to baseline risk category. Comparison of cumulative CR between triple and dual therapy within each baseline risk category, revealing a significant association between triple therapy and CR in the high-risk group (**C**), but not in the low- (**A**) or moderate-risk (**B**) groups. CR: complete renal response

Finally, the treatment effects were examined within each risk stratum by comparing the cumulative CR rates between the triple and dual therapies (Fig. 4). In the low- and moderate-risk groups, the cumulative CR rates did not differ between the treatment strategies (95.7% vs. 97.5%, *p* = 0.28; 81.8% vs. 63.2%, *p* = 0.45, respectively). In contrast, among high-risk patients, triple therapy was associated with a significantly higher cumulative CR rate than dual therapy (56.7% vs. 27.1%, *p* = 0.01).

To determine whether this association was independent of baseline disease characteristics, multivariable Cox proportional hazards regression analysis was performed in the high-risk group (Table 3). After adjustment for baseline UPCR, disease duration, and eGFR, triple therapy remained independently associated with achievement of CR (adjusted HR 3.13, 95% CI 1.17–8.73, *p* = 0.02).

**Table 3:** Multivariable Cox proportional hazards regression analysis of CR in high-risk LN

	**HR (95% CI)**	***p***
Triple therapy	3.13 (1.17–8.73)	0.02
UPCR (g/gCr)	0.61 (0.06–5.58)	0.67
Disease duration	0.99 (0.99–1.00)	0.28
eGFR (mL/min/1.73 m^2^)	0.94 (0.34–2.56)	0.91

*UPCR* Urinary protein-to-creatinine ratio; eGFR, glomerular filtration rate

## Discussion

In this study, we found that baseline risk stratification using established prognostic factors summarized in the 2025 EULAR recommendations identified subgroups in which triple therapy was associated with higher CR rates. Nephrotic-range proteinuria and history of LN flares emerged as the strongest predictors of induction failure. By integrating these major risk factors with additional prognostic variables, patients could be categorized into low-, moderate-, and high-risk groups, each exhibiting distinct trajectories of remission induction. Notably, the observed association between triple therapy and CR was confined to the high-risk group, whereas no incremental association was observed in patients classified as low or moderate risk.

Recent international recommendations, including the 2024 ACR and 2025 EULAR, emphasize the early use of combination regimens to improve renal outcomes in LN [8, 9]. At the same time, these recommendations acknowledge that treatment decisions should be individualized and that less intensive regimens remain appropriate for selected patients without poor prognostic features. However, translating these recommendations into routine clinical practice has been challenging, because explicit guidance on how to operationalize prognostic factors for treatment selection has been limited. In this context, our findings suggest a potential framework for the future evaluation of risk-adapted treatment strategies.

Importantly, the classification of prognostic factors into "major" and "minor" was developed specifically for this study based on multivariate analysis and should not be interpreted as a categorization proposed by the 2025 EULAR recommendations. The designation of "minor" reflects the absence of an independent association with CR after multivariable adjustment in this cohort and does not imply lesser clinical importance.

Among the EULAR 2025 prognostic factors, nephrotic-range proteinuria and previous LN flares were independent predictors of induction failure. These observations are consistent with prior studies showing that heavy baseline proteinuria is strongly associated with delayed or incomplete renal response and adverse long-term renal outcomes [16–18]. Similarly, a history of renal flares reflects cumulative structural damage and has been repeatedly linked to reduced responsiveness to subsequent therapy and an increased risk of chronic kidney disease progression [19, 20]. Our findings support the clinical relevance of these prognostic factors for identifying patients with high risk of poor renal outcome.

By contrast, individual minor prognostic factors did not independently predict remission failure when present in isolation. Patients with a single minor risk factor had CR rates comparable with those without prognostic factors. This finding should be interpreted with caution, as the number of patients harboring only one minor risk factor was small (10.6%) and EULAR-defined prognostic factors rarely occur in isolation in real-world practice. Instead, these factors tend to cluster, and their prognostic relevance appears to emerge through the cumulative burden. Indeed, the CR rates declined markedly in patients with at least one major risk factor combined with multiple additional prognostic factors. Moreover, patients with more prognostic factors exhibited the poorest outcomes, further supporting the cumulative clinical significance of the EULAR 2025 framework.

A key observation of this study was that the benefits of triple therapy were restricted to high-risk patients. In this subgroup, triple therapy significantly improved the cumulative CR rates compared to dual therapy, whereas no such benefit was observed in low- or moderate-risk patients. Importantly, the association remained statistically significant after adjustment for baseline renal function, proteinuria, and disease duration, indicating that it was not fully accounted for by these measured baseline characteristics. These findings also suggest that risk-adapted treatment escalation may achieve a more favorable balance between efficacy, safety, and treatment burden. This approach aligns with the broader concept of precision immunosuppression, which intensifies therapy in patients most likely to benefit, while avoiding overtreatment in those with more favorable prognostic profiles [21, 22].

This study has certain limitations. This was an observational study based on a medical record review and a single-center study, and the treatment allocation was not randomized, raising the possibility of residual confounding factors. Therapeutic strategies and supportive care evolved over the extended study period, which may have influenced the outcomes, despite consistent definitions of response. Treatment selection was at the discretion of the treating physicians and may have been influenced by the perceived disease severity, introducing the possibility of confounding by indication. Despite adjustment for clinically relevant covariates, residual confounding factors cannot be completely excluded. Therefore, the observed association between triple therapy and CR should not be interpreted as evidence of a causal treatment effect. In addition, the cohort consisted exclusively of Japanese patients, which potentially limits the generalizability of our findings to other populations. Prospective validation in independent and ethnically diverse cohorts is essential to confirm the applicability and external validity of this risk-stratification model. Our risk stratification model was developed using established baseline prognostic factors and therefore did not incorporate immunological biomarkers, comprehensive histopathological scoring, or dynamic disease activity markers during follow-up. Future studies that integrate these parameters may improve the predictive performance of the model. The proposed risk stratification model and all performance analyses were derived and evaluated within the same observational cohort and should, therefore, be regarded as exploratory and hypothesis-generating instead of independently validated. Furthermore, the subgroup analyses evaluating treatment effects included a relatively small number of patients and should, therefore, be regarded as exploratory. External validation using independent prospective cohorts is required before this approach can be applied in routine clinical practice. Residual confounding factors cannot be completely excluded because of the observational design based on medical record review, nonrandomized treatment allocation, and the fact that not all baseline prognostic factors were included in the multivariate Cox model. In addition, several subgroup analyses included a relatively small number of patients, which may have reduced the stability and precision of the Kaplan–Meier and Cox regression estimates. Therefore, these findings should be interpreted with caution and require confirmation in larger independent cohorts.

## Conclusions

In conclusion, combining established prognostic factors into a risk stratification model may facilitate clinically meaningful risk stratification in patients with LN. This approach identified subgroups in which triple therapy was associated with higher CR rates. However, because this risk stratification model was derived and evaluated within the same observational cohort, whether treatment should be tailored according to this strategy requires prospective validation in independent cohorts before implementation in routine clinical practice.

## Supplementary Information


Supplementary Material 1


## Data Availability

All data supporting the findings of this study are available within the paper and its Supplementary Information.

## Abbreviations

ACEi: Angiotensin-converting enzyme inhibitor
ACR: American College of Rheumatology
AI: Activity index
ARB: Angiotensin II receptor blocker
BMI: Body mass index
CI: Chronicity index
CR: Complete renal response
eGFR: Estimated glomerular filtration rate
EULAR: European Alliance of Associations for Rheumatology
HCQ: Hydroxychloroquine
HR: Hazard ratio
ISN/RPS: International Society of Nephrology/Renal Pathology Society
LN: Lupus nephritis
MMF: Mycophenolate mofetil
OR: Odds ratio
pCYC: Pulsed cyclophosphamide
SLE: Systemic lupus erythematosus
SLEDAI: Systemic Lupus Erythematosus Disease Activity Index
TAC: Tacrolimus
UPCR: Urinary protein-to-creatinine ratio
