# Supplementary material for: Early risk stratification in lupus nephritis based on established prognostic factors: association with outcomes under triple therapy
Source: Arthritis Res Ther. 2026 Jul 31;28:158. doi: 10.1186/s13075-026-03876-w (PMC13523271; doi:10.1186/s13075-026-03876-w)
Supplement: Supplementary file 1 [file 13075_2026_3876_MOESM1_ESM.pdf]

SFigure 1

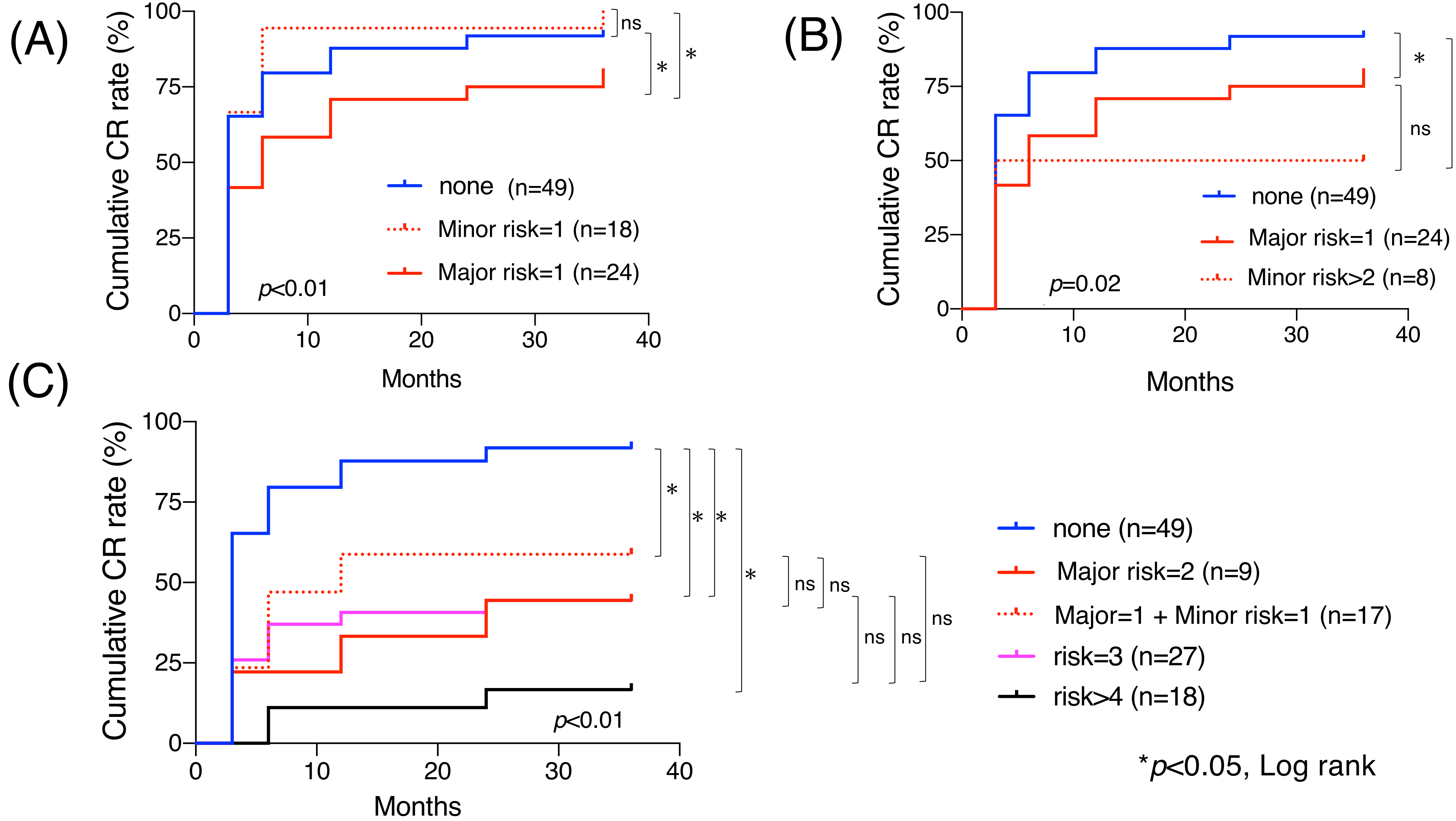

*p<0.05, Log rank
